# Generation of a rabbit single-chain fragment variable (scFv) antibody for specific detection of *Bradyrhizobium* sp. DOA9 in both free-living and bacteroid forms

**DOI:** 10.1371/journal.pone.0179983

**Published:** 2017-06-27

**Authors:** Nguyen Xuan Vu, Natcha Pruksametanan, Witsanu Srila, Watcharin Yuttavanichakul, Kamonluck Teamtisong, Neung Teaumroong, Nantakorn Boonkerd, Panlada Tittabutr, Montarop Yamabhai

**Affiliations:** 1 Molecular Biotechnology Laboratory, School of Biotechnology, Institute of Agricultural Technology, Suranaree University of Technology, Nakhon Ratchasima, Thailand; 2 Biofertilizer & Plant–Microbe Interaction Research Unit, School of Biotechnology, Institute of Agricultural Technology, Suranaree University of Technology, Nakhon Ratchasima, Thailand; 3 The Center for Scientific and Technological Equipment, Suranaree University of Technology, Nakhon Ratchasima, Thailand; US Naval Research Laboratory, UNITED STATES

## Abstract

A simple and reliable method for the detection of specific nitrogen-fixing bacteria in both free-living and bacteroid forms is essential for the development and application of biofertilizer. Traditionally, a polyclonal antibody generated from an immunized rabbit was used for detection. However, the disadvantages of using a polyclonal antibody include limited supply and cross-reactivity to related bacterial strains. This is the first report on the application of phage display technology for the generation of a rabbit recombinant monoclonal antibody for specific detection and monitoring of nitrogen-fixing bacteria in both free-living form and in plant nodules. *Bradyrhizobium* sp. DOA9, a broad host range soil bacteria, originally isolated from the root nodules of *Aeschynomene americana* in Thailand was used as a model in this study. A recombinant single-chain fragment variable (scFv) antibody library was constructed from the spleen of a rabbit immunized with DOA9. After three rounds of biopanning, one specific phage-displayed scFv antibody, designated bDOA9rb8, was identified. Specific binding of this antibody was confirmed by phage enzyme-linked immunosorbent assay (phage ELISA). The phage antibody could bind specifically to DOA9 in both free-living cells (pure culture) and bacteroids inside plant nodules. In addition to phage ELISA, specific and robust immunofluorescence staining of both free-living and bacteroid forms could also be observed by confocal-immunofluorescence imaging, without cross-reactivity with other tested bradyrhizobial strains. Moreover, specific binding of free scFv to DOA9 was also demonstrated by ELISA. This recombinant antibody can also be used for the study of the molecular mechanism of plant–microbe interactions in the future.

## Introduction

*Bradyrhizobium* is one of the soil bacteria that can fix nitrogen in symbiosis with specific legumes and convert nitrogen gas into ammonia to be used as fertilizer in plants. *Bradyrhizobium* sp. DOA9 was originally isolated from the root nodules of *Aeschynomene americana* in Thailand. This strain was classified as *B*. *yuanmingense* based on multilocus DNA sequence analysis of 16S rRNA and housekeeping genes (*dnaK*, *recA*, and *glnB*) [1]. DOA9 is a divergent *nod*-containing strain that can fix nitrogen under free-living conditions. Interestingly, this strain was able to colonize and efficiently nodulate the roots of most plants ranging from the Dalbergioid, Millettioid, and Robinioid tribes [2]. Due to its broad host range ability, DOA9 is an interesting strain to be further developed as a multi-purpose inoculant as well as to explore its mechanism of interaction with plants at the molecular level.

An efficient systematic procedure for bacterial cell identification and monitoring is needed to maintain the reliability of biofertilizer. The detection of *Rhizobium* can be done by many methods including microbial, serology, and DNA techniques [3]. Serology is a simple and rapid technique when compared to microbiological or DNA-based techniques [4]. Both polyclonal and monoclonal antibodies have been used for enzyme-linked immunosorbent assay (ELISA), immunoagglutination, or immunofluorescence detection of target strains in culture broth or in nodules, as well as for monitoring the persistence of inoculant in the soil [5–7]. Serological technique can also be used as a tool to study the key determinants involved in legume-rhizobium nodulation. Monoclonal antibodies were used as analytical probes to elucidate the structural differences among lipopolysaccharide (LPS) molecules in *R*. *leguminosarum* strain 3841 in comparison with LPS-defective mutant derivatives [8]. Polyclonal antibodies raised against LPS from bradyrhizobial strains that nodulate peanut have been shown to cross-react with the peanut specific bradyrhizobia but not *B*. *japonicum* [9]. This result suggested the presence of certain common LPS antigenic determinants among the strains. Therefore, it is possible that polyclonal antibody could have cross-reactivity with other indigenous bacteria sharing similar antigenic determinant presence in the soil, creating difficulty in result interpretation [5].

Application of monoclonal antibodies for identification of a rhizobial strain has been done for the first time against a commercial inoculant of *Rhizobium trifolii* 162X95 [9]. This monoclonal antibody was used in an indirect ELISA to differentiate strain 162X95 from indigenous strains collected from the Appalachian region where this strain was isolated, and also used for detecting the bacteroid from crushed nodules with a strong immunological reaction. However, the traditional method for monoclonal production is quite complicated. Moreover, monoclonal antibodies may not be the ideal method for bacterial identification if those monoclonal antibodies bind with nonspecific or invariant antigenic determinant molecule among bacteria. Therefore, the efficient method of production and screening the monoclonal antibody that bind with the specific antigenic molecule of each rhizobium are required.

The development of antibody engineering techniques has provided ways to produce antibodies in many formats [10]. Compared to all antibody-based methods, phage-display technology is a highly attractive alternative method for the production of monoclonal antibodies against diverse antigens [11]. The phage-display technique allows isolation of antibodies directly from diverse repertoires of antibody genes [12]. These antibody genes are expressed on the surface of filamentous bacteriophages as fusion proteins [13]. Therefore, antibody phage-display technology permits the immortalization of monoclonal antibody genes. This technique also permits improvement of the antibody to suit various applications, such as enhancing stability, increasing binding affinity, or fusion with various reporter genes [14].

From various recombinant antibody formats, a single-chain fragment variable (scFv) is particularly favorable. An scFv molecule consists of a variable region of heavy chains and light chains that are linked together by a flexible linker. The advantages of scFv molecules are small size, long storage, and the ability to engineer and produce at large scale [15]. Phage-display technology is an efficient system for the generation of scFv molecules with desired binding affinity and specificity for many antigen types [16]. Phage display antibody technology has been successfully used to isolate specific peptides and monoclonal antibodies against various targets including different pathogenic bacteria, viruses, parasites and mycotoxins [17–19]. In the aspect of rhizobial research, phage-display technology has been used for the identification of extracytoplasmic proteins from *B*. *japonicum* by taking advantage of phagemid that lacks its indigenous signal peptide to select for the genes encoding signal peptides from rhizobia and display its protein molecule on the phage [20]. However, generation of a specific antibody against beneficial soil bacteria by using phage display technique has never been reported. In this study, phage-display antibody technology has been successfully employed for the production of a specific scFv antibody against *Bradyrhizobium* strain DOA9. This work will serve as a basis for the development of an efficient detecting reagent for agricultural applications as well as for the study of plant–microbe interactions in the future.

## Materials and methods

### Materials

All chemical reagents were molecular biology grade. *Escherichia coli* TG1 was obtained from MRC, Cambridge, and used for cloning and amplification of phages. *E*. *coli* HSM174 pLysS (Novagen) was used for protein expression. The anti-M13/HRP and His prob-HRP detection kits were purchased from Amersham-Pharmacia Biotech (Uppsala). Protein L peroxidase-HRP, anti-rabbit IgG-FITC and Calcofluor white stain were purchased from Sigma. *Bradyrhizobium* strain DOA9 and other bradyrhizobia were isolated from *A*. *americana* in our laboratory [1], while the reference strains of *B*. *diazoefficiens* USDA110 and *Bacillus subtilis* 168 were obtained from the School of Biotechnology, SUT.

### Ethics statement

This study was carried out in strict accordance with the recommendations in the Ethical Principles and Guidelines for the Use of Animals provided by National Research Council of Thailand (NRCT). The protocol was approved by the Committee on the Ethics of Animal Experiments of Suranaree University of Technology and the Agricultural Research Development Agency (Public Organization), Thailand (contract number CRP5507010890). Euthanasia was done using carbon dioxide (CO_2_), and finally completed by cervical dislocation method. Verification of complete euthanasia was done by feeling the chest between thumb and forefinger to ensure that the heart was not beating and there was no blink reflex by touching the eyeball. All efforts were made to minimize suffering and provide the rabbit with the painless death.

### Preparation of antigen samples

#### Pure culture antigen

The *Bradyrhizobium* sp. strain DOA9 (DOA9) was maintained as previously described [3]. Cells were centrifuged at 3,400 x g at 4°C for 10 min and the supernatant was discarded. The pellet was washed three times with sterilized saline buffer, then the cell concentration was adjusted to 10^9^ cells/ml. The cell suspension was treated by boiling in a water bath for 1 h to denature flagella and other protein antigens. The total protein was determined by the Bradford assay [21]. Normally, approx. 100μg/ml of total protein from each preparation was obtained. Finally, merthiolate was added to achieve a final concentration of 1: 10,000 as preservation. The stock of pure antigen was stored at −20°C until use.

#### Nodule antigen

Seeds of siratro (*Macroptilium atropurpureum*) were surface-sterilized and germinated according to the standard method [4]. The germinated seeds were planted in Leonard’s jars and inoculated with the tested *Bradyrhizobium* at 10^9^ cells/seed. All plants were supplemented with N-free medium and grown in a light room for one month. Then, nodules were collected from plants and washed with sterile distilled water. Nodules were stored over silica gel at room temperature. Prior to analyses, nodules were revived by distilled water for 1–2 h. The total protein of bacterial strains inside the nodules was difficult to predict as the number of bacteroid was varied among the nodule. However, it can be roughly estimated that the amount of total protein was in range of 90–120 μg/ml.

### Immunization

Healthy young White New Zealand rabbits (6–12 months) were immunized with prepared DOA9 cell antigen through intravenous injection every day for 5 days. After 2 weeks of immunization, the antibody titer was determined by agglutination reaction using antigen prepared from boiled DOA9 culture [3]. The two-fold dilutions of antibody were prepared and tested with antigen as previously described [4]. Once the titer was higher than 1: 1,600, 20–30 ml of blood was collected and centrifuged at 4,500 rpm for 15 min to obtain polyclonal antibody serum. Serum was stored at −20°C until use. Finally, rabbits were sacrificed and spleens were removed directly to liquid nitrogen and kept at −80°C. Note that intravenous injection (IV) is an unusual route for polyclonal antibody production in rabbit, but commonly used for the subsequent boosting. IV injection was done in this study to avoid the side effect of the adjuvant that occurred when administered by intramuscular injection (IM) [22].

### Construction of recombinant scFv library

The procedure for the construction of the immunized rabbit recombinant scFv library was conducted as described previously [23]. Total RNA was extracted from rabbit spleens by TRIzol reagent (Invitrogen) according to the manufacturer’s instructions. Then, cDNA templates were prepared by using MMuLV reverse transcriptase enzyme (NEB) using a mixture of oligo-dT_18_ and random hexamer primers. A total of 29 PCR reactions were used to amplify the variable regions of heavy-chain, κ light-chain, and λ light-chain (V_H_, V_L_κ, and V_L_λ) genes. All of the primers used are listed in Table 1. The heavy-chain 5' primers were designed to include an *Sfi* I site, while the light-chain 3' primers included a *Not* I site. Light-chain 5' primers were designed to include part of the linker region (Gly4Ser)_3_ and were compatible with the heavy-chain 3' primers. The assembly PCR reaction contained an equal molar mixture of the pooled heavy (V_H_) DNA and pooled light (V_L_κ and V_L_λ) gene repertoire. The final step created a full-length scFv gene repertoire from the second PCR by PCR amplification in the presence of pull-through primers. This PCR extended the scFv gene from the *Sfi* I and *Not* I sites flanking scFv genes, using the following primers: PTfw (5'-CCT TTC TAT GCG GCC CAG CCG GCC ATG GCC-3') and PTrv (5'-CAG TCA TTC TCG ACT TGC GGC CGC ACG-3'). The scFv DNA fragments were digested with *Not* I and *Sfi* I and ligated into a linearized pMOD1 vector [23]. The ligation reaction was transformed into competent *E*. *coli* TG1 cells by electroporation (Maxim Biotech Inc, USA), using an Eppendorf 2510 electroporator (Eppendorf). After overnight incubation at 37°C, colonies were collected, mixed with glycerol, and stored at −80°C. The library size was determined by serial plating on TYE (10 g Tryptone, 5 g Yeast extract, 8 g NaCl, 15 g Bacto-agar dissolved in 1 L of H_2_O) plates, supplemented with 1% glucose and 100 μg/ml amplicillin and incubated overnight at 37°C. The diversity of the library was analyzed by *BstN* I digestion and random automated DNA sequence analysis.

**Table 1:** Oligonucleotide primers used for the construction of the immunized rabbit phage library.

Primer	Sequence
rV_H_5'*Sfi*I	1. 5' GCC CAG CCG GCC atg gcc CAG TCG GTG GAG GAG TCC RGG 3'
	2. 5' GCC CAG CCG GCC atg gcc CAG TCG GTG AAG GAG TCC GAG 3'
	3. 5' GCC CAG CCG GCC atg gcc CAG TCG YTG GAG GAG TCC GGG 3'
	4. 5' GCC CAG CCG GCC atg gcc CAG SAG CAG CTG RTG GAG TCC GG 3'
	5. 5' GCC CAG CCG GCC atg gcc CAG TCG CTG GAG GAG TCC GGG GGT 3'
rV_H_3'link	1. 5' gcc aga acc gcc tcc ccc act ccc tcc gcc acc CGATGGGCCCTTGGTGGAGGCTGARGAGAYGGTGACCAGGGTGCC 3'
	2. 5' gcc aga acc gcc tcc ccc act ccc tcc gcc acc GACTGAYGGAGCCTTAGGTTGC 3'
rVL5'link-κ	k1. 5' agt ggg gga ggc ggt tct ggc gga ggt ggg tcg GAG CTC GTG MTG ACC CAG ACT CCA 3'
	k2. 5' agt ggg gga ggc ggt tct ggc gga ggt ggg tcg GAG CTC GAT MTG ACC CAG ACT CCA 3'
	k3. 5' agt ggg gga ggc ggt tct ggc gga ggt ggg tcg GAG CTC GTG ATG ACC CAG ACT GAA 3'
	k4. 5' agt ggg gga ggc ggt tct ggc gga ggt ggg tcg GCT CAA GTG CTG ACC CAG AC 3'
	k5. 5' agt ggg gga ggc ggt tct ggc gga ggt ggg tcg GMC MYY GWK MTG ACC CAG ACT CC 3'
rVL5'link-λ	l1. 5' agt ggg gga ggc ggt tct ggc gga ggt ggg tcg GAG CTC GTG CTG ACT CAG TCG CCC TC 3'
	l2. 5' agt ggg gga ggc ggt tct ggc gga ggt ggg tcg CAG CCT GTG CTG ACT CAG TCG 3'
rVL3'NotI-κ	k1. 5' cag tca ttc tcg act tGC GGC CGC ACG TTT GAT TTC CAC ATT GGT GCC 3'
	k2. 5' cag tca ttc tcg act tGC GGC CGC ACG TAG GAT CTC CAG CTC GGT CCC 3'
	k3. 5' cag tca ttc tcg act tGC GGC CGC ACG TTT GAC SAC CAC CTC GGT CCC 3'
rVL3'NotI-λ	L1. 5' cag tca ttc tcg act tGC GGC CGC GCC TGT GAC GGT CAG CTG GGT CCC 3'
	L2. 5' cag tca ttc tcg act tGC GGC CGC RCC TGT GAC GGT CAG CTG GGT CC 3'
m/rPtfw	5’ cct ttc tat gcG GCC CAG CCG GCC atg gcc 3’
m/rPtrv	5’ cag tca ttc tcg act tGC GGC CGC 3’

S = G/C, R = G/A, K = G/T, M = A/C, Y = C/T, W = A/T, H = A/C/T, B = C/G/T, V = A/C/G, D = A/G/T, N = A/T/G/C

### Biopanning against *Bradyrhizobium* sp. strain DOA9

Biopanning of the immunized rabbit scFv recombinant antibody library against strain DOA9 was conducted according to the protocol described previously [23]. The selection was done in an immunotube (Nunc) containing 20 μg of antigen (total protein of boiled *Bradyrhizobium*) in 400 μl of 100 mM NaHCO_3_ at pH 8.5. The immunotube was then blocked to avoid non-specific binding of phage particles with 2% (w/v) skimmed milk in phosphate-buffered saline (MPBS). The phage library containing 10^12^ PFU of phages in 300 μl of 2% MPBS was added to the tube and incubated at room temperature for 1 h with rotation and 1 h on a bench at room temperature without rotation. Unbound phages were removed by washing three times with PBS containing 0.1% (v/v) Tween 20 (PBST) and twice with PBS. The bound phages were eluted by trypsinization and low pH condition using acidic elution buffer (50 mM glycine-HCl pH 2.0). The recovered phages from both elutions were amplified in *E*. *coli* TG1 cells. To determine output titer, serial dilutions of eluted phages were plated out on 2×YT plates containing 100 μg/ml of ampicillin and 1% glucose. Three rounds of biopanning (affinity selection) were performed. For each round of selection, the specificity of randomly picked individual phage scFv clones was determined by monoclonal phage ELISA.

### Preparation of phage-displayed scFvs for monoclonal phage ELISA

To amplify individual phage clones for phage ELISA, single phage colonies were randomly picked from the 2xYT plates containing 100 μg/ml of ampicillin and 1% glucose and grown in wells of a 96-well microculture plate (Nunc) containing 100 μl 2×YT plus 100 μg/ml ampicillin and 1% (w/v) glucose. After overnight incubation at 37°C, a small inoculum (5 μl) from each well was transferred to a second 96-well plate containing 400 μl of 2×YT plus 100 μg/ml ampicillin and 1% (w/v) glucose in each well. The transferred plate was incubated with shaking at 37°C for 3 h, and later phages were rescued by adding 10^10^ PFU of helper phage to each well. Then, the plate was incubated at 37°C for 1 h before spinning at 4,500 rpm for 10 min. Then, the supernatant was discharged and the pellet was suspended in 400 μl of 2×YT containing 100 μg/ml ampicillin and 50 μg/ml kanamycin, and cultured at 30°C for 20 h with shaking (250 rpm). The next day, the plate was centrifuged at 4,500 rpm for 10 min, and 100 μl of the supernatant containing phages was used in monoclonal phage ELISA. This protocol can be scaled up to amplify phage in a larger scale. Occasionally, the phage particles are purified and concentration by PEG precipitation accordingly to published protocols [24]. The numbers of phage clones in culture supernatant are varied, depending on each phage clone. Normally approx. 10^7−9^ pfu/μl of phage could be obtained in culture supernatant; while approx. 10^11−12^ pfu/μl of phage could be obtained after PEG precipitation.

### Detection of DOA9 strain by phage ELISA

#### Pure culture

Phage ELISA was done as previously described [24]. A total of 8 μg of boiled culture antigens i.e., *Bradyrhizobium* sp. DOA9 [DOA9], other bradyrhizobia (*Bradyrhizobium* sp. SUTN9-2 [SUTN9-2], *Bradyrhizobium* sp. SUTN1-12 [SUTN1-12], and *Bradyrhizobium diazoefficiens* USDA110 [USDA110]), and *B*. *subtilis* 168 were diluted in sodium carbonate buffer to achieve 5 μg total protein per well. These bacterial samples were immobilized on a 96-well ELISA plate (MicroWell^™^, Nunc). After blocking with MPBS and washed, 4x10^12^ pfu of phage (100 μl of phage supernatant containing 4x10^13^ pfu/ml) was added into each well. The phage clone 3C1, isolated from a naïve human scFv antibody library (against aflatoxin B1) was used as a negative phage control. Bound phages were detected with mouse anti-M13 bacteriophage conjugated with horseradish peroxidase (HRP) (Amersham-Pharmacia Biotech), according to the manufacturer’s protocol (1:5,000 dilution in 1xblocking buffer). Rabbit polyclonal antibody (1:7,500 dilution, from at least 1.0 mg/ml stock) against DOA9 was used as a positive control. Bound rabbit IgG was detected with 1:8,000 dilution of protein A-HRP (Invitrogen). Color reactions were developed using ABTS (2, 2-azino-di-3-ethyl-benzothiazoline-6-sulfonate) peroxidase substrate (Amresco) containing 0.05% H_2_O_2_. Detection was done by measuring the absorbance at 405 nm in an ELISA plate reader (Sunrise, TECAN, Austria). The assay was performed in triplicate.

#### Nodule sample

To prepare plant nodules for phage ELISA, five nodules from various rhizobial strains for each replication were crushed and gently ground in a small sterilized mortar containing 1 ml of sodium carbonate buffer. Then, 200 μl of the nodule suspension was added to each well of an ELISA plate (Nunc). The assay was performed in triplicate as described for pure culture samples.

### Detection of DOA9 strain by immunofluorescence analysis

The immunofluorescence analysis was performed according to the protocol described by Somasegaran and Hoben [4] with some modifications. For pure culture, the bacteria spotted on the slide were incubated with 10 μl of polyclonal antibody (1: 7,500 dilution) or PEG-precipitated phage (10^12^ Pfu) for 1 h. The phage clone 3C1, which binds to aflatoxin B1, was used as a non-related phage control. The slide was rinsed twice with PBS and incubated with 10 μl of 1:100 dilution of secondary FITC-conjugated anti-rabbit IgG antibody (for polyclonal antibody) or anti-M13-FITC (for phage antibody) in a moist chamber for 1 h at room temperature. Unbound proteins were removed by rinsing and washing with PBS and mounted in 40% glycerol under a cover slip. The slide was examined under a confocal fluorescence microscope (Nikon A1). The nodule was prepared according to the method of cytological analysis of legume nodule [25]. A section of 30 μm thickness was generated using a microtome and placed on slides. Then, sections were incubated in PBS containing Calcofluor white M2R (Sigma) to a final concentration of 0.01% (w/v) for staining of the plant cell wall. After rinsing with PBS, the procedure was done as described for pure culture samples.

### DNA sequence analysis

Plasmids containing selected scFv inserts were prepared using a plasmid purification kit as suggested by the manufacturer (Mini Prep; Qiagen). Automated DNA sequencing was performed by Macrogen (Seoul, Korea) using -96gIII (5'-CCC TCA TAG TTA GCG TAA CG-3') and Yamo_5 (5'-CAG GAA ACA GCT ATG ACC-3') primers. The DNA sequences were then compared with the rabbit germline sequence via IMGT, the International ImMunoGeneTics information system (http://www.imgt.org). The amino acid sequences were translated using ExPASy. The three-dimensional structure was predicted using the Phyre^2^ program [26], and visualized and annotated using PyMOL software (www.pymol.org).

### Production of soluble scFv antibody

The gene of positive scFv antibody clone (bDOA9rb8) were sub-cloned into pET27b vectors (Novagen) between *Nco* I and *Not* I sites, and transformed into *E*. *coli* HSM 174 pLysS for expression. *E*. *coli* harboring the recombinant plasmid was grown at 30°C in 5 ml of M9ZB media containing 50 μg/ml kanamycin and 2% (w/v) glucose. Then, a 1: 100 dilution of each overnight culture was inoculated into M9ZB media supplemented with 2% w/v glucose and 50 μg/ml kanamycin and cultured with shaking at 30°C for 4 h. After that, the cells were centrifuged at 16°C, 5,000 rpm for 15 min followed by re-suspension with M9ZB media containing 1% (w/v) glycerol, 50 μg/ml kanamycin, and 1 mM isopropylthiogalactoside (IPTG; Emresco, USA). After continuing incubation at 16°C for 20 h with shaking, the cell pellet was collected by centrifugation at 16°C, 8,000 rpm for 15 min. The secreted antibody could be found in the supernatant and periplasmic space of the *E*. *coli* cells. Extraction of scFv from periplasm was done according to published protocol [24]. A hexahistidine tag was used for detection and purification. The scFv fragments were purified by one-step Immobilized Metal Affinity Chromatography (IMAC), using Ni^2+^ ions immobilized on resin by covalent linkage to nitrilotriacetic acid (NTA) in accordance with the manufacturer’s protocol (Qiagen GmbH). The column was washed two times with 50 ml of wash buffer (20 mM Tris–HCl buffer, pH 8.0 and 150 mM NaCl) containing 20 mM imidazole. Ni–NTA bound enzyme was eluted with 250 mM imidazole in the same buffer. Purity of the eluted scFv was analyzed by 12% SDS-PAGE analysis (staining with Coomassie brilliant blue), and by Westernblot analysis. The binding specificity of soluble scFv antibodies was evaluated by ELISA with both types of antigen, i.e., pure culture and nodule samples. The procedure was similar with phage ELISA. Soluble scFv antibodies were detected with His probe-HRP (Thermo Fisher Scientific) (1: 5000 from a 1 ug/ml stock). For Westernblot analysis, HRP substrate from Amersham ECL Prime Western Blotting Detection Reagent was used.

## Results and discussion

### Construction and biopanning of a rabbit scFv antibody library against *Bradyrhizobium* sp. strain DOA9

**Fig 1:**
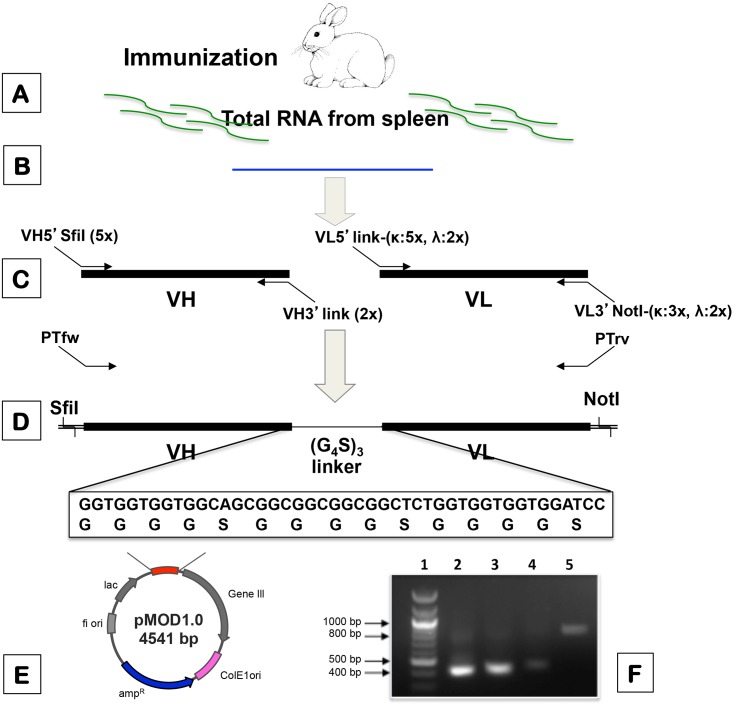
Schematic outline of the procedure for the construction of the immunized rabbit scFv library. Once the antibody titer of immunized rabbit, as determined by agglutination method, was higher than 1:1600, the total RNA was extracted from spleen (**A**). Then, cDNA was synthesized by using a mix of random hexamers and oligo-dT_18_ (**B**). The locations of all PCR primers on the two variable regions genes are indicated (**C**). The list of all primers used for the construction of the library is given in Table 1. The first PCR step comprised 29 PCR reactions for amplification of V_H_ and V_L_ gene repertoire. Then, equal amounts of V_H_ and V_L_κ, and V_H_ and V_L_λ were assembled together via a (G_4_S)_3_ linker sequence by overlap extension, followed by pull-through PCR steps (**D**). The amplified full-length scFv repertoire fragments were then cloned into a pMOD1.0 vector (see full-size map in Supporting information S2 Fig), between *Sfi* I and *Not* I sites, before being transformed into *E*. *coli* TG1 (**E**). The agarose gel (inset) illustrates an example of DNA fragments from various steps; lane 1: 100 bp DNA ladder (NEB, USA); lane 2: V_L_λ; lane 3: V_L_κ; lane 4: V_H_; and lane 5: assembled scFv fragments (**F**). Please note that even the PCR products of V_H_ obtained from the PCR reactions were quite low as shown as a faint band in the lane 4 of the gel, we were able to use them to generate assemble products of scFv genes by pull-through PCR as shown in lane 5.

A schematic overview of the procedure for library construction is illustrated in Fig 1. A small immunized rabbit scFv library containing 3.5 × 10^6^ independent clones was constructed. Restriction fragment analysis of inserts from 20 randomly picked clones indicated that 85% of inserts contained full-length scFv genes (see Supporting information S1 Fig). There were no unique *BstN* I digestion patterns in the inserted clones. Significant enrichment of the specific scFv phage antibody was observed after each round of affinity selection as indicated by higher percentage of output ratio after each round of biopanning (Table 2). In each round of affinity selection (biopanning), 192 phage clones (two 96-well ELISA plates) were randomly picked and amplified to confirm the binding to DOA9 by phage ELISA (skim milk was used as negative controls). Therefore, a total of 576 phage clones were screened. From a total of 576 clones tested, two positive phage clones that showed high binding signal were obtained. These two clones, designated RB9 and RG8, were chosen for further study.

**Table 2:** Selective enrichment of scFv antibodies during the biopanning process.

Round	Amount of antigen (μg)^a^	Input (PFU)^b^	Output (PFU)^b^	Output ratio^c^ %
1^st^	20	1.0 × 10^12^	2.57 × 10^4^	2.6 × 10^−6^
2^nd^	15	1.0 × 10^12^	1.72 × 10^5^	1.7 × 10^−5^
3^rd^	10	1.0 ×10^12^	1.02 × 10^6^	1.0×10^−4^

Three rounds of biopanning were performed. In each round the target was reduced to increase the chance to obtain strong binders.

^a^The amount of antigen is the total protein of boiled pure culture of *Bradyrhizobium* sp. DOA9, determined by Bradford assay as described in the Materials and method section.

^b^PFU is plaque forming unit, indicating the number of phage clones added into immunotube and the eluted bound phage obtained from each round of biopanning.

^c^Output ratio (%) = (phage output number × 100)/(phage input number)

### Specific binding of phage-displayed recombinant scFv against *Bradyrhizobium* sp. DOA9

**Fig 2:**
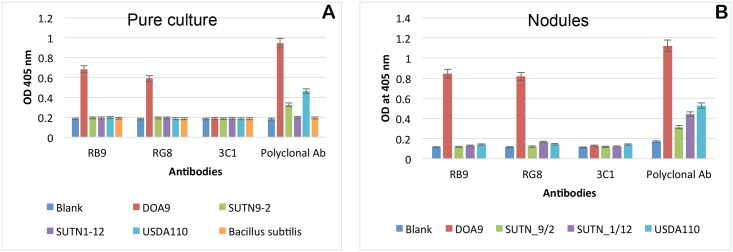
Specific binding of selected phage-displayed scFv clones. Phage ELISA results of the binding of scFv antibodies against DOA9 and other antigens in pure culture (**A**) and plant nodules (**B**) are shown. The two clones of phage-displayed rabbit scFv, i.e., RB9 and RG8, could bind specifically to only strain DOA9 but not to other *Bradyrhizobium* strains and *Bacillus subtilis* 168. Phage-displayed human 3C1 scFv antibody was used as a negative control in this assay. The average OD_405_ nm values and standard errors from triplicate wells are shown. Note that in panel B, there was no bacillus nodule because it can’t form nodule in plant roots.

Binding properties of phage-displayed scFv and rabbit polyclonal antibodies were observed by ELISA as shown in Fig 2 for both free-living (A) and bacteroid (B) forms of bacteria. Both polyclonal and phage-displayed scFv antibodies showed a clear binding signal to DOA9. No signal was observed with the negative control phage, 3C1 (specific mycotoxin-binding phage [18]). Most importantly, the two phage-displayed scFv clones (RB9 and RG8) showed no cross-reactivity with other related bradyrhizobial strains, i.e., USDA110, SUTN9-2, and SUTN1-12, or *Bacillus* sp., while some cross-reactivity with non-related strains could be observed when using a polyclonal antibody. These results indicate that the binding specificity of the phage-displayed monoclonal antibody is higher than that of a polyclonal antibody.

### Amino acid sequence analysis of specific phage clones

**Fig 3:**
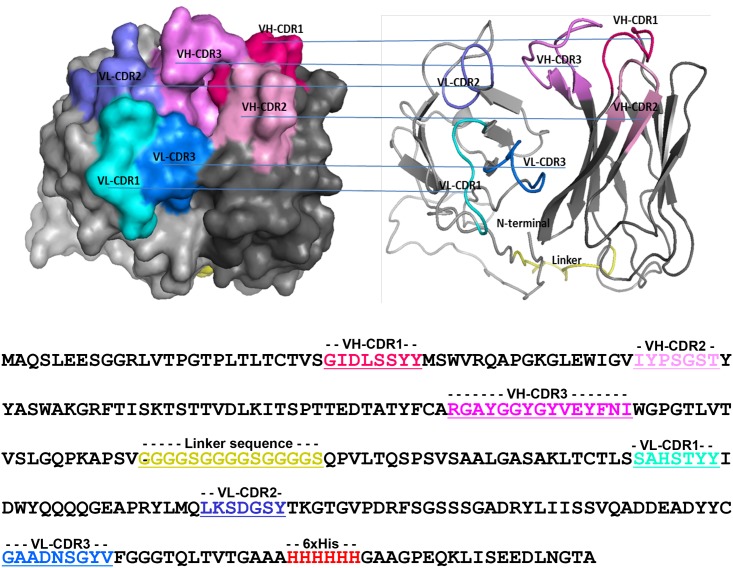
Amino acid sequence and 3D structure of isolated anti-DOA9 rabbit scFv antibody. The primary amino acid sequence of the clone bDOA9rb8 is shown at the bottom. The flexible linker (G_4_S)_3_ that joins V_H_ and V_L_ segments is indicated. The three complementarity-determining regions (CDRs) are underlined. The 3D structure was done by Phyre^2^ software, using the structure of an scFv antibody in complex with an analogue of the main immunogenic region of the acetylcholine receptor (PDB code: 1F3R) as templates. The 3D structures in both space-filling and ribbon models with CDRs of V_H_ and V_L_ domains are indicated.

The DNA sequences of the positive phage clones, i.e., RB9 and RG8, were analyzed by automated DNA sequencing. The result indicated that the sequences of RB9 and RG8 were identical. Amino acid sequence analysis of the specific phage clones using IMGT^®^ software (http://www.imgt.org/IMGT_vquest/vquest) revealed that the rabbit scFv antibody belongs to immunoglobulin VH1 for heavy chain and VL λ for light chain. This recombinant antibody was designated bDOA9rb8. The amino acid sequence and the 3D structure of the antibody are shown in Fig 3.

The identity of the antigen on the surface of DOA9 that interacts with the selected antibody in this study is yet to be identified. This interacting molecule must be present in both free bacteria and bacteroid form, according to the phage ELISA result shown in Fig 2. *Bradyrhizobium* sp. usually contain extracellular polysaccharide (EPS), capsular polysaccharides (CPS) or K-antigen, flagella proteins or H-antigen, and lipopolysaccharide (LPS) or O-antigen [27]. In our study, the DOA9 cells were boiled before injection into the rabbit or immobilization onto an immunotube or ELISA plate. The K- and H-antigens, which are heat labile, would have been destroyed by heat. Therefore, the candidate epitope of this selected antibody is likely to be present on the O-antigen, which contains strain-specific oligosaccharide repeating units that are heat resistant. Monoclonal antibodies have been used to study the O-antigen component of LPS from *R*. *leguminosarum* strain 3841 [28] and the core component of LPS and lipid A [8]. The results from these studies reveal that LPS contain several molecular structures that represented both strain-invariant molecules and conserve structures among the closely related rhizobia. Therefore, the cell surface molecules of strain DOA9 that can interact with the antibodies could be one of several components of LPS. The fact that the selected clone bDOA9rb8 from this study showed specificity to only DOA9 but not related bradyrhizobial strains indicated that this antibody might interact with the strain specific molecular structure on the somatic antigen (O-antigen) of this bacterium.

### Serial dilution experiment of phage-displayed recombinant scFv antibody against *Bradyrhizobium* sp. DOA9

**Fig 4:**
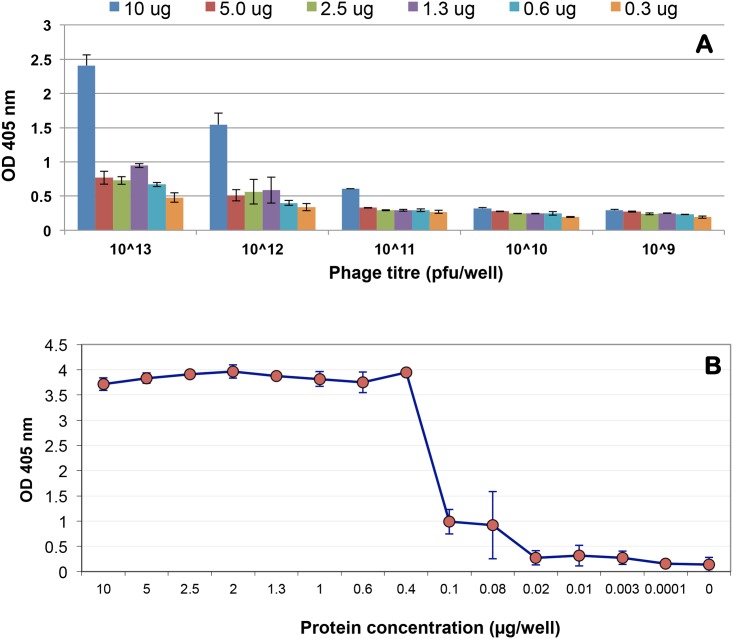
Antibody characterization via dilution series. (**A**) A checkerboard titration experiment of phage-displayed scFv clone bDOA9rb8. Various amount of phage particles were prepared by 10-fold dilution series starting from 10^13^, 10^12^, 10^11^, 10^10^, and 10^9^ pfu/well. Each dilution of phage antibody was used to detect different amount of boiled DOA9 antigen at the protein concentration of 10, 5.0, 2.5, 1.3, 0.6 and 0.3 μg/well of total protein, determined by Bradford assay. Each reaction was done in triplicate. The phages were purified by PEG precipitation and re-suspended in PBS buffer at a concentration of 10^11^ pfu/μl. Bound phage was detected by anti-M13 HRP, using ABTS as color reagent. The Y-axis indicated the OD value and the SD measured from triplicate wells. (**B**) Detection limit of phage displayed scFv clone bDOA9rb8 against boiled DOA9. The 5-fold dilution series of boiled DOA9 protein antigen were prepared in sodium carbonate buffer starting from the protein concentration of 10, 2, 0.4, 0.08, 0.02, 0.003 and 0.00 μg/well. The ELISA was performed by using the optimum concentration of phage antibody (10^12^ pfu/well), determined from panel A. The average OD_405_ nm values and standard errors from triplicate wells are shown.

To determine the binding property of phage-displayed scFv clone bDOA9rb8, a two-component serial dilution phage ELISA or a checkerboard experiment was conducted as shown in Fig 4A. Various amount of boiled DOA9, ranging from 10 to 0.3 μg was immobilized on wells of 96-well ELISA plate and incubated with various amount of phage clones ranging from 10^13^ to 10^9^ pfu/well. To conduct this experiment, the phage particles were concentrate by PEG precipitation and re-suspended in PBS buffer as described in the Materials and methods section. The antigen used in this study was pure culture of DOA9 that was boiled as described in the methods section. The amount of the target antigens that was added into each well of ELISA plates was based on total protein, determined by Bradyford assay. This target was used for immunization, biopanning, and phage ELISA as shown in Fig 2. The result obtained from the serial dilutions experiment indicated that phage-dislayed scFv at the concentraion of 10^12^ pfu/well was an optimal concentration for detection in the direct ELISA formats. This phage concentration was used for immunofluorescence and limit of detection (LOD) analyses in the next steps.

The binding property of selected phage-display scFv to boiled DOA9 antigen was further investigate to determine the limit of detection as illustrated in panel B. The result showed that there was a sharp drop in ELISA signal when the total protein of target antigen droped from 0.4 to 0.1 μg/welll while there were no reduction of binding signal when the total proteins were reduced from 10–0.4 μg/well. This result suggested that the estimation of the boiled bacterial antigen based on total protein content might not be accurate, as it is likely that the target antigens that interact with the selected antibody are polysaccharide moiety in the lipopolysacchardies (LPS) molecule [29] as discussed above. Further investigation on the structure of the core oligosaccharide or lipid A component of *Bradyrhizobium* LPS that interacts with this antibody will be interesting to fully understand the function and biosynthesis of surface molecules on the cell wall of these bacteria [8].

### Production of free scFv antibody fragments

**Fig 5:**
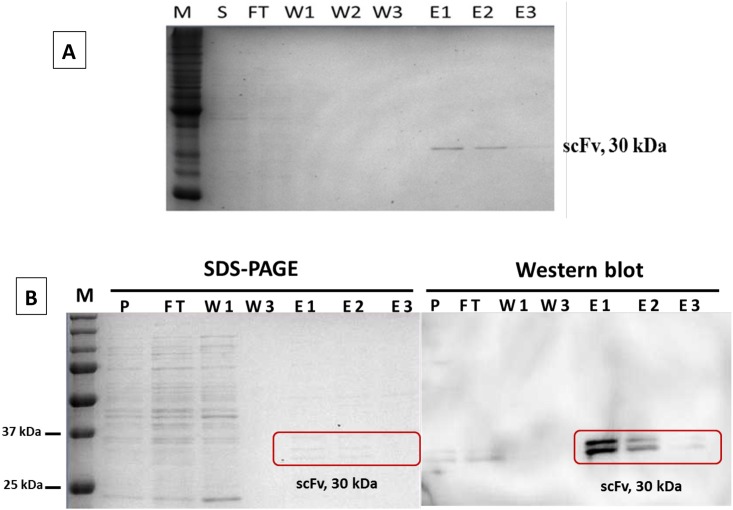
SDS-PAGE and Westernblot analysis of purified soluble scFv antibody. (**A**) The soluble scFv antibody against *Bradyrhizobium* sp. strain DOA9 clone bDOA9rb8 was purified from culture supernatant by IMAC. Lane M: protein molecular weight marker; lane S: culture supernatant input; lane FT: flow-through fraction; lanes W1, W2, and W3 indicate the three wash fractions; lanes E1, E2, and E3 are the three elution fractions. The soluble scFv antibody of approx. 30 kDa can be found in elution fractions 1 and 2. This scFv antibody was used to study the binding specificity in the next step. (**B**) SDS-PAGE and Western blot analysis of free scFv clone bDOA9rb8 obtained from periplasmic extracts.

To demonstrate the binding of free scFv antibody independent of phage particle, the soluble form of the rabbit scFv antibody against strain DOA9 (bDOA9rb8) was produced by sub-cloning the scFv gene into pET27b vector and expressed in *E*. *coli* HSM 174 for protein expression. The recombinant antibody scFv was over-expressed under the induction of IPTG and the secreted recombinant antibody could be purified from the culture supernatant or periplasmic space by one-step immobilized metal affinity chromatography (IMAC). The results of SDS-PAGE showed that the recombinant rabbit scFv antibody against *Bradyrhizobium* strain DOA9 with a size of about 30 kDa could be successfully purified from the culture supernatant (Fig 5A) or periplasm (Fig 5B) of *E*. *coli*, using Ni-NTA resin as demonstrated in Fig 5. The antibody band on an SDS-PAGE of the antibody prepared from periplasmic space was further confirmed by westernblot analysis (Fig 5B). Double bands indicated incomplete cleavage for signal peptide from scFv molecule [24]. Routinely, approx. 2–5 mg/ml of total protein could be obtained from a shake-flask preparation in the laboratory. The antibody obtained from this study was used in the serial dilutions experiments in the next step.

**Fig 6:**
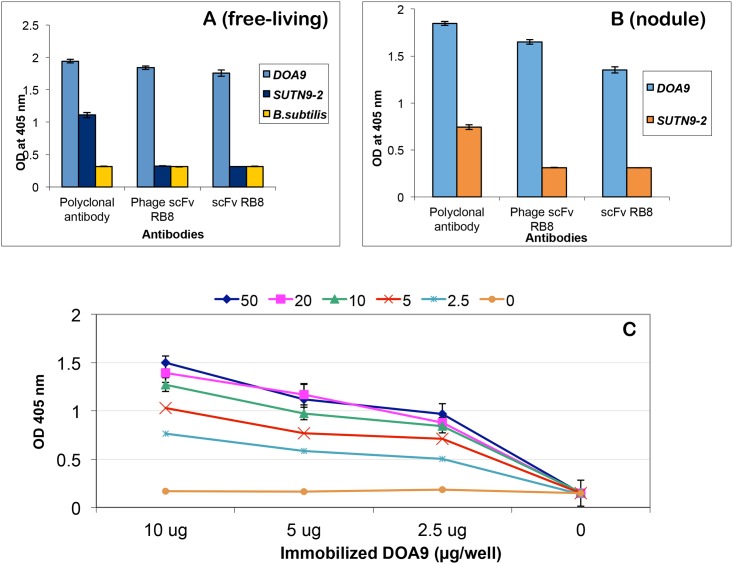
Binding property of free scFv against strain DOA9. ELISA results of soluble scFv antibodies against bacterial targets in both free-living (**A**) and bacteroid form (**B**) in plant nodule are illustrated. Rabbit polyclonal antibody and Phage-display scFv were also used in the assay for comparison. Values are the mean of triplicate wells. Error bars show the standard deviation for each set of data. Panel **C** illustrates binding properties of free scFv against DOA9 in a checkerboard titration experiment. Serial dilutions of soluble scFv antibody ranging from 50–0 μg were add into wells of ELISA plated immobilized with various concentration of boiled DOA9, ranging from 10 to 0 μg of total protein. The Y-axis indicated the OD value and the SD measured from triplicate wells.

The binding property of free scFv clone bDOA9rb8 was demonstrated by ELISA (Fig 6). To test for the binding specificity, secreted recombinant scFv from culture broth was tested against DOA9 and closely related bradyrhizobial strains, i.e., SUTN9-2, and *Bacillus* sp in both pure culture or free living (A) and bacteroid form in plant nodules (B). Note that there was no *Bacillus* in panel B because this bacterium can’t form nodule in plant root. These results demonstrate that free scFv antibodies retain specific binding activity against both forms of the bacteria. To estimate the binding strength of the recombinant scFv, a checkerboard titration experiment was performed as shown in Fig 6C. In this serial dilution experiment, recombinant scFv was purified from perimplasmic extract of *E*. *coli* expressing recombinant scFv gene. Various concentration of scFv antibody ranging from 50–0 μg/well was incubated with 2-fold serial dilutions of boiled DOA9 samples from 10–0 μg/well. The ELISA signals indicated that as low as 2.5 μg/well of antibody could be used to detect as low as 2.5 μg/well of total protein of boiled DOA9.

This is the first report on the generation of a specific recombinant scFv monoclonal antibody against bradyrhizobial members using phage-display antibody technology. Further optimization for large-scale antibody expression in an *E*. *coli* system as well as stability analysis or antibody engineering to suit field applications are necessary to achieve real benefits for the agricultural sector in the future. In order to achieve this goal, various issues have to be considered. These include the application of different methods of antibody engineering to improve the antibody property such as binding affinity and robustness [30]. The antibody can also be engineered into various formats to suit various purposes such as conversion into whole IgG or fusion with reporter protein [31]. The engineered antibody can then be use in agglutination test, as convenient one-step detection probe, or assembled with lateral flow strip test or nanoparticle biosensor, as appropriated [32]. Another important consideration is the optimization of the bioprocess for the production and purification of recombinant antibody from *E*. *coli* or other appropriate expression systems [33].

### Potential applications of phage-displayed scFv clone bDOA9rb8

In order to demonstrate the utility of isolated phage-displayed scFv that was generated in this study, two different detection strategies were employed. Immunofluorescence technique is very informative and can be used to detect DOA9 in both nodule and free-living forms, allowing investigation of the mechanism of nodule organogenesis as well as for monitoring the free-living bacteria along the biofertilizer production process. However, the immunofluorescence procedure is highly sophisticated, expensive, and can’t be applied in the field. We, therefore; demonstrated the application of phage-displayed scFv bDOA9rb8 for a simple detection assay of whole cell bacteria in free-living form using ELISA format. This assay is inexpensive and can be readily applied for agriculture uses. Experimental results that demonstrate the potential application of phage-display scFv generated in this study are described below.

#### Immunofluorescence staining of *Bradyrhizobioum* sp. DOA9 in both pure culture and nodule forms

**Fig 7:**
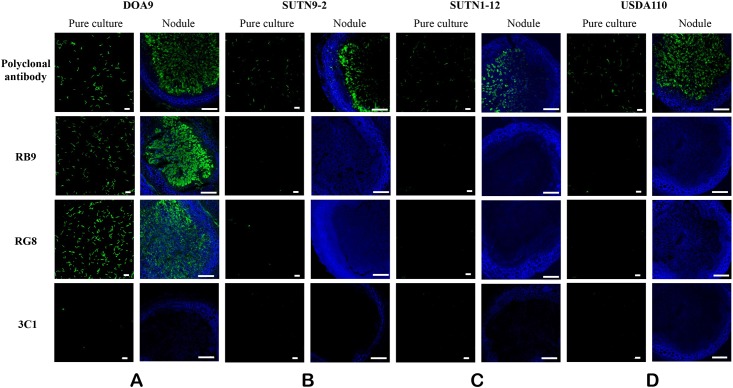
Confocal microscopy images of immunofluorescence staining for *Bradyrhizobium* sp. prepared from pure culture and plant nodules. Immunofluorescence staining of various bacterial targets in both pure culture and nodule forms are shown. The bacteria tested include *Bradyrhizobium* sp. strains DOA9 (**A**), SUTN9-2 (**B**), SUTN1-12 (**C**), and USDA110 (**D**). The bacterial samples were stained with various antibodies, i.e., rabbit polyclonal antibody, two clones of phage-displayed rabbit scFv, i.e., RB9 and RG8, which contained identical DNA sequence. Phage-displayed scFv antibody clone 3C1 that bind aflatoxin B1 was used as a negative control. Green spots indicated the green fluorescent staining of phage display scFv or polyclonal antibody, using secondary antibody conjugated to FITC. Plant cell walls were stained with a blue fluorophore (Calcofluor white M2R) and emitted blue color. The bacteroid are shown as green spots inside a blue plant cavity. Scale bar is 10 μm at 80× magnification, and 100 μm at 10× magnification for pure culture and nodule samples, respectively.

Immunofluorescence analysis was performed to confirm the specific binding of the phage-displayed scFv and to visualize the morphology of the bacteroid form in the plant nodule directly under the microscope. The two clones of phage-displayed rabbit scFv, i.e., RB9 and RG8, which contained identical DNA sequence, could bind specifically to only strain DOA9 obtained from both pure culture and nodules, but not to other related *Bradyrhizobium* strains, including SUTN9-2, SUTN1-12, or USDA110 (Fig 7). No fluorescence staining was detected with the negative controls, including no phage, no polyclonal antibody, and non-related scFv antibody (mycotoxin-specific phage clone, 3C1). The plant cell wall was indicated by the blue color of Calcofluor staining. The two antibody clones showed no cross-reactivity against non-related *Bradyrhizobium* strains. These results are the opposite to those obtained from using the polyclonal antibody, which showed strong cross-reactivity against all non-related bradyrhizobial strains tested, especially those in the plant nodules (Fig 7 top panel). The specific binding results were shown in duplicate as the phage clones RB9 and RG8 contained exactly the same DNA sequence, which subsequently designated, bDOA9rb8.

**Fig 8:**
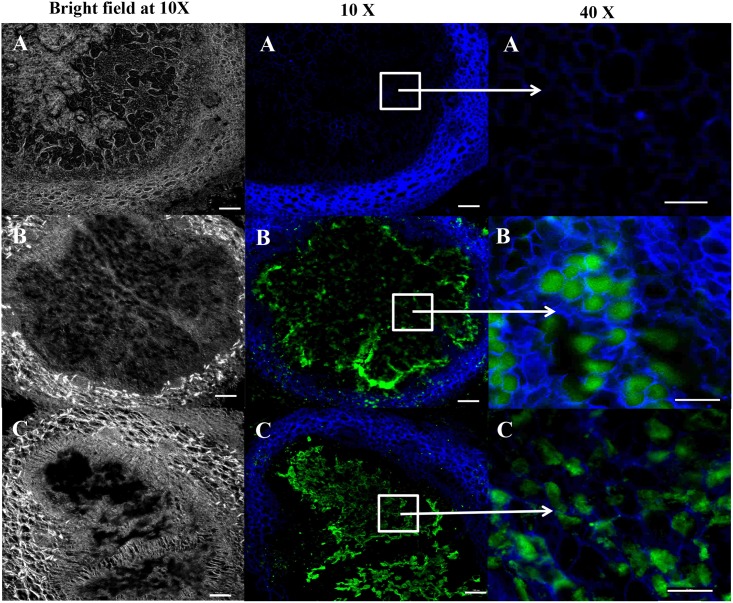
Investigation of the morphology of symbiotic nodule using phage-displayed scFv clone bDOA9rb8. Bright field and immunofluorescence staining images from confocal microscopy of bacteriod form of *Bradyrhizobium* sp. DOA9 in plant nodules at different magnifications are shown. Sample in **A** was detected with negative phage clone 3C1; **B** rabbit polyclonal antibody, or **C** the phage-displayed scFv clone bDOA9rb8 from this study. Blue color indicated the plate cell wall that was stained with blue fluorophore. Scale bar is 100 μm at 10X magnification and 50 μm at 40X magnifications.

The advantage of using immunofluoresecence technique to visualize the morphology of the symbiosis nodule, which is essential to study the molecular mechanism of plant-microbe interactions, is demonstrate in Fig 8. When compare with the bright field, the morphology of symbiosis nodule can be observed. Traditionally, polyclonal antibody is used to stain the nodule; therefore, the result of immunofluorescence staining using polyclonal antibody was also shown for comparison. As shown in the 40x magnification, the pattern of the staining between phage and polyclonal antibody is different. The superior specificity of phage scFv when compared to rabbit antibody made it attractive for certain purposes such as for the investigation of double occupancy of the nodules or competition between rhizobium strains in nodule formation [34].

The ability to apply phage-displayed scFv antibody to study the morphology of bacteriod form will allow scientists to detect and identify the specific microorganism or antigen simultaneously in ecological condition [35, 36]. Therefore, the phage-displayed scFv antibody that was obtained from this study could be used to investigate the symbiosis interaction, nodule organogenesis and occupancy as well as using for detection the pure culture along the biofertilizer production process. Moreover, identification of the common cell surface molecule of the bacteria and bacteroid that can interact with this antibody would be interesting and useful for the study of the molecular mechanism of plant–microbe interaction as well.

#### Limit of detection of phage-display scFv antibody against whole DOA9 cells

**Fig 9:**
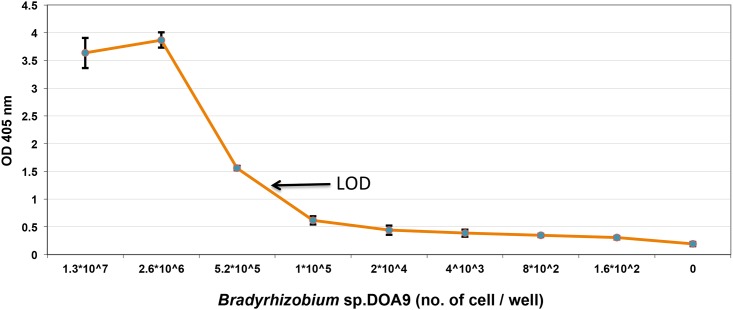
Determination of limit of detection (LOD) of whole cell DOA9 by phage ELISA using phage-displayed scFv bDOA9rb8. The 5-fold dilution series of whole cell DOA9 was prepared in sodium carbonate buffer starting from 1.3×10^7^, 2.6×10^6^, 5.2×10^5^, 1×10^5^, 2×10^4^, 4×10^3^, 8×10^2^, 1.6×10^2^, and 0 cell/well. Phage-displayed scFv bDOA9rb8 was added at 10^12^ pfu/μl/well and detected by Phage ELISA as described. The lowest number of cells that gave the good signal from ELISA (indicated by arrow) was the detection limit of *Bradyrhizobium* sp. DOA9 in form of whole cell antigen.

An efficient, non-GMO based method for identification of selected bacterial strains is necessary for successful application of biofertilizer, both for the preparation of culture inoculants and for monitoring in the field [6]. Up to now, many techniques based on polyclonal antibody–antigen reactions for the analysis of rhizobial inoculants have been developed. However, the problem with using a polyclonal antibody is cross-reactivity with other rhizobial strains within the same species. This cross-reactivity may also extend to other rhizobial biovars or species and sometimes even to members of other genera of bacteria [37]. The cross-reactivity problem can be overcome by pre-absorption of a given polyclonal antibody with other related strains or using monoclonal antibody instead. The usefulness of phage-displayed scFv clone bDOA9rb8, generated in this study, for biofertilizer application was demonstrated in an ELISA-based method as illustrated in Fig 9. A serial dilution assay of whole *Bradyrhizobium* DOA9 from pure culture inoculant was performed to demonstrate the limit of detection of DOA9 cell numbers using ELISA-based method. The optimal phage scFv concentration as determined from checkerboard titration, i.e., 10^12^ pfu/μl/well, was used in this study. A series of 5-fold dilutions of *Bradyrhizobium* DOA9 whole cells were directly immobilized onto wells of microtiter plates. This Phage ELISA result indicated that the limit of detection (LOD) of this assay was approx. 4–5 x10^5^ cells.

The limit of detection of phage-displayed bDOA9rb8 scFv antibody obtained in this study is sufficient for monitoring most of the biofertilizer inoculants that are commonly used in the field; however, determination of possible false positives and negatives must be further investigated before real application [3].

## Conclusions

In conclusion, a recombinant scFv antibody that can bind specifically to *Bradyrhizobium* sp. strain DOA9 was successfully generated from an immunized rabbit phage-display scFv antibody library. The recombinant scFv antibody, designated bDOA9rb8, in both soluble and phage-displayed formats could bind to the bacteria in both free-living and bacteroid form inside the plant nodules. This antibody showed high specificity with DOA9 and no cross-reactivity against other tested related bradyrhizobial strains. The potential applications of phage-display scFv against DOA9 were demonstrated by phage ELISA and immunofluorescence assay.

This work was supported by Suranaree University of Technology and the SUT-PhD Scholarship Program for ASEAN and by the Office of the Higher Education Commission under the NRU project of Thailand. It was also supported by the National Research Council of Thailand and by the Agricultural Research Development Agency (Public Organization), Thailand CRP5507010890. The funders had no role in study design, data collection and analysis, decision to publish, or preparation of the manuscript.

## Supporting information

S1 Fig Restriction fragment analysis of 20 randomly picked phage clones. The isolated phagemids (about 500ng DNA/reaction) were digested with *Nco* I and *Not* I. The result showed that 17/20 clones (85%) had inserted fragment. The clones that have no inserts are labeled in red.
(TIF)

S2 Fig Map of pMOD1.0 vector. This vector was generated in Molecular Biotechnology Laboraotry at SUT as described in Pansri P, Jaruseranee, N., Rangnoi, K., Kristensen, P., Yamabhai, M. A compact phage display human scFv library for selection of antibodies to a wide variety of antigens. BMC Biotechnol 2009; 9:6.
(TIF)
