# Supplementary figures and images for: Generation of a rabbit single-chain fragment variable (scFv) antibody for specific detection of Bradyrhizobium sp. DOA9 in both free-living and bacteroid forms
Source: PLoS One. 2017 Jun 27;12(6):e0179983. doi: 10.1371/journal.pone.0179983 (PMC5487062; doi:10.1371/journal.pone.0179983)

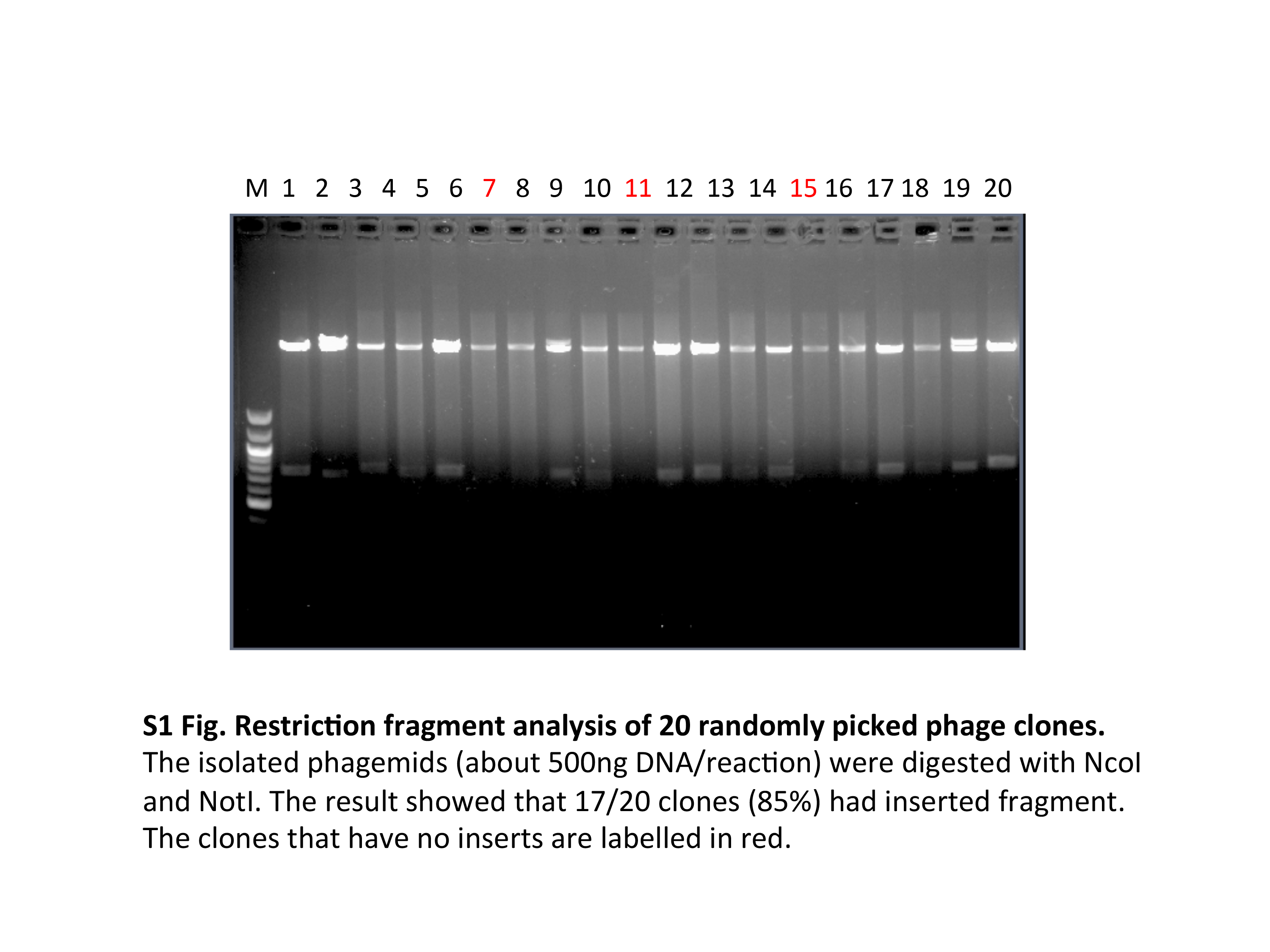

Supplement: S1 Fig — The isolated phagemids (about 500ng DNA/reaction) were digested with Nco I and Not I. The result showed that 17/20 clones (85%) had inserted fragment. The clones that have no inserts are labeled in red. (TIF) [file pone.0179983.s001.tif]

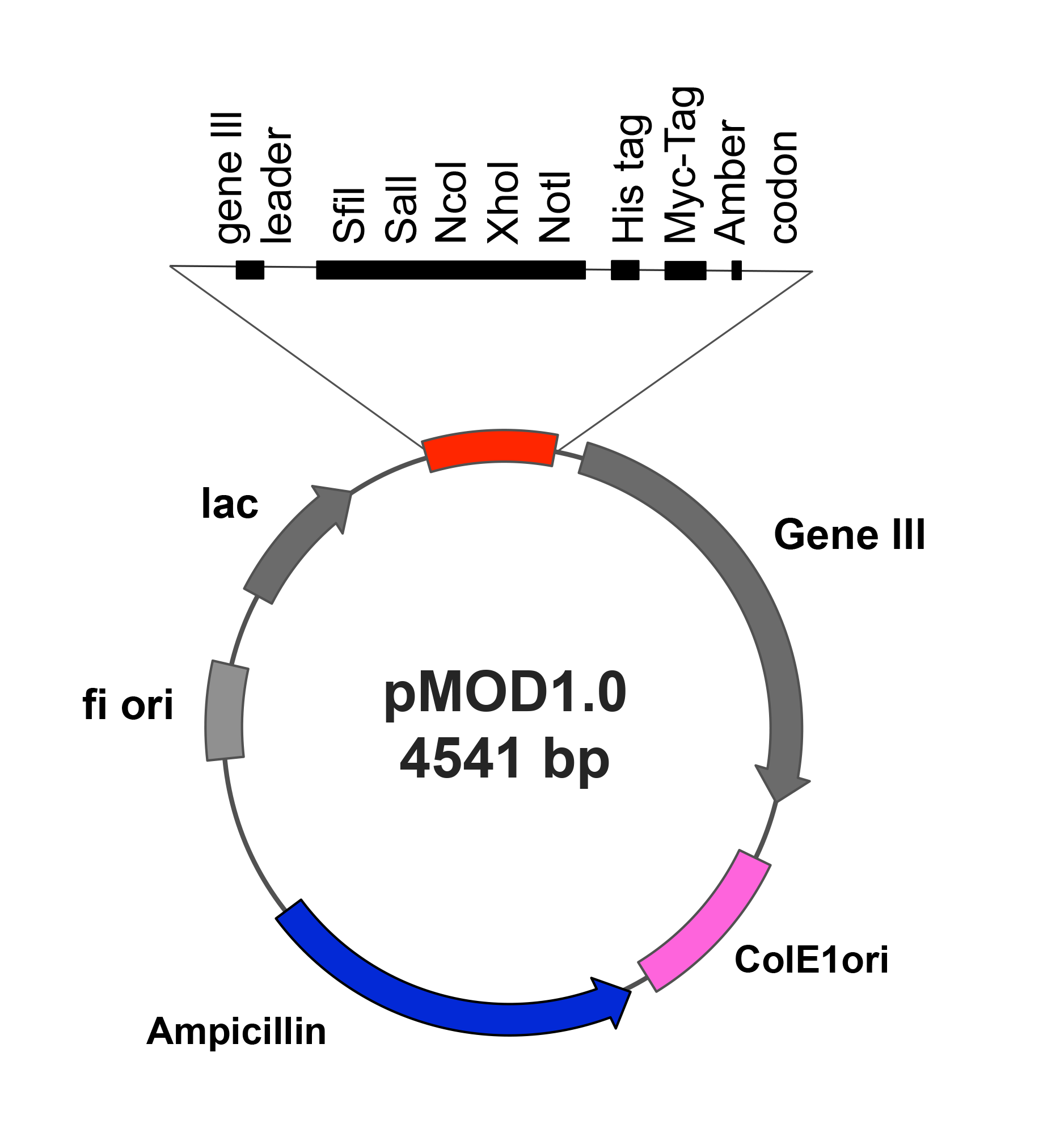

Supplement: S2 Fig — This vector was generated in Molecular Biotechnology Laboraotry at SUT as described in Pansri P, Jaruseranee, N., Rangnoi, K., Kristensen, P., Yamabhai, M. A compact phage display human scFv library for selection of antibodies to a wide variety of antigens. BMC Biotechnol 2009; 9:6. (TIF) [file pone.0179983.s002.tif]
